# Predator Cue and Prey Density Interactively Influence Indirect Effects on Basal Resources in Intertidal Oyster Reefs

**DOI:** 10.1371/journal.pone.0044839

**Published:** 2012-09-07

**Authors:** A. Randall Hughes, Kelly Rooker, Meagan Murdock, David L. Kimbro

**Affiliations:** 1 Coastal and Marine Laboratory, Florida State University, St. Teresa, Florida, United States of America; 2 Department of Biology, Bridgewater College, Bridgewater, Virginia, United States of America; University of Gothenburg, Sweden

## Abstract

Predators can influence prey abundance and traits by direct consumption, as well as by non-consumptive effects of visual, olfactory, or tactile cues. The strength of these non-consumptive effects (NCEs) can be influenced by a variety of factors, including predator foraging mode, temporal variation in predator cues, and the density of competing prey. Testing the relative importance of these factors for determining NCEs is critical to our understanding of predator-prey interactions in a variety of settings. We addressed this knowledge gap by conducting two mesocosm experiments in a tri-trophic intertidal oyster reef food web. More specifically, we tested how a predatory fish (hardhead catfish, *Ariopsis felis*) directly influenced their prey (mud crabs, *Panopeus* spp.) and indirectly affected basal resources (juvenile oysters, *Crassostrea virginica*), as well as whether these direct and indirect effects changed across a density gradient of competing prey. Per capita crab foraging rates were inversely influenced by crab density, but they were not affected by water-borne predator cues. As a result, direct consumptive effects on prey foraging rates were stronger than non-consumptive effects. In contrast, predator cue and crab density interactively influenced indirect predator effects on oyster mortality in two experiments, with trait-mediated and density-mediated effects of similar magnitude operating to enhance oyster abundance. Consistent differences between a variable predator cue environment and other predator cue treatments (no cue and constant cue) suggests that an understanding of the natural risk environment experienced by prey is critical to testing and interpreting trait-mediated indirect interactions. Further, the prey response to the risk environment may be highly dependent on prey density, particularly in prey populations with strong intra-specific interactions.

## Introduction

Predators can influence prey abundance and traits by direct consumption, as well as by non-consumptive effects of visual, olfactory, or tactile cues [1], [2], [3]. Both changes in prey abundance due to consumption and changes in prey behavior due to predator presence can influence prey foraging and energy acquisition [3], [4], [5], [6]; thus, separating consumptive effects (CEs) and non-consumptive effects (NCEs) is not always a trivial task. But because predator effects can also cascade down to basal resources [4], [7], [8], [9], understanding the effects of independent and combined CEs and NCEs on prey foraging can be critical for understanding food web dynamics across multiple trophic levels [3].

Specific predator characteristics (e.g., predator identity, foraging modality) can be important for determining the strength of non-consumptive predator effects [10], [11], [12]. For example, variation in predator hunting strategy can influence the strength of NCEs: sit-and-wait predators often elicit stronger NCEs than actively hunting predators, although the mechanisms underlying this effect are not completely understood [10], [11]. In addition, diet breadth within and across predator species can influence the strength of predator effects generally [13], [14], [15], and there is some evidence that it also influences the strength of non-consumptive effects [12]. For instance, the top predator in intertidal oyster reef communities, the oyster toadfish, benefits juvenile oyster survivorship primarily by affecting the behavior of, rather than consuming, the intermediate mud crab consumers [12], [16], [17]. The overall strength of NCEs, however, is diminished when the toadfish is replaced by the omnivorous blue crab that consumes both oysters and mud crabs [12].

In addition to effects of predator identity or foraging characteristics, temporal variation in predation risk is likely important to the strength of NCEs [18], [19], [20], [21], [22], [23]. Predators may exhibit predictable diel movements based on day-night or tidal cycles, and thus experimental manipulations with constant predator cues may over-estimate the strength of NCEs in these systems. Conversely, a non-linear relationship between predator cues and prey behavior (see, e.g., [20]) can result in strong NCEs even in relatively ‘safe’ environments with low temporal exposure to predation risk, as predicted by the predation risk allocation hypothesis [18]. Although there have been a handful of experimental tests of the predation risk hypothesis [20], [21], [22], [23], most have focused on prey behavior during low or high risk pulses, and they have not evaluated how these behavioral responses translate to prey resource abundance over time.

Prey density can vary due to consumptive predator effects or from processes unrelated to predation (e.g., competition for resources, recruitment), and such variation in prey numbers may influence the strength of non-consumptive predator effects on prey and basal resources. For example, the non-consumptive effects of bird predators on grasshoppers varied by grasshopper density due to the presence of a trade-off between survival and reproduction with increasing density [24]. Such effects of prey density on the strength of NCEs may be more common in systems in which prey foraging rates are strongly influenced by interference interactions among conspecifics (e.g., strong competition for resources [24], [25]).

We conducted two mesocosm experiments to quantify predator effects on both their prey (mud crabs, *Panopeus* spp.) and basal resources (juvenile oysters, *Crassostrea virginica*) in a tri-trophic intertidal oyster reef food web. By using a novel predator species (the hardhead catfish, *Ariopsis felis*) in a different geographic location (the northeastern Gulf of Mexico) than previous related studies (e.g., [12], [16], [17], we indirectly examined the context dependency of predator effects in oyster reef communities. We first tested the relative importance of predator consumptive (quantified by simulated predation) and non-consumptive (quantified by exposure to water-borne predator cues) effects on prey per capita foraging rates, and resulting density-mediated (DMII) and trait-mediated (TMII) indirect effects on basal resource abundance (see Methods for definitions and calculations of effect sizes). In a subsequent experiment, we tested whether non-consumptive predator effects are independently and interactively influenced by prey density and predator cue environment (constant vs. variable).

## Results

**Figure 1:**
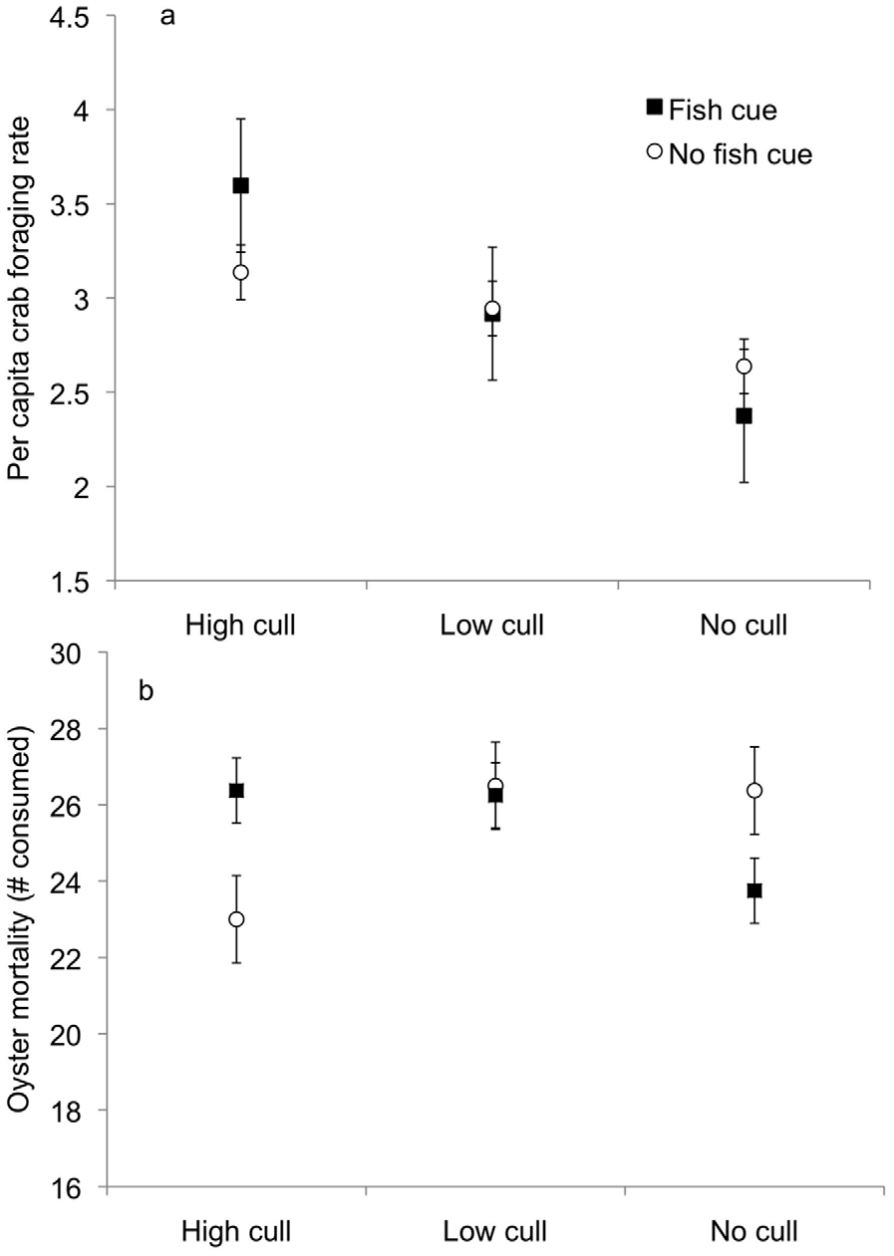
The effects of predator cue and mud crab removal rate on (a) mud crab per capita foraging rate and (b) the number of juvenile oysters consumed during Experiment I. There was no effect of predator cue on per capita mud crab foraging rates, but we show the predator cue treatments separately for comparison with panel b. Closed squares indicate catfish cue is present at high tide; open circles indicate catfish cue is absent at high tide. Symbols represent means(±SE).

In Experiment I, the crab removal treatment best explained direct predator effects on per capita crab foraging rates, regardless of predator cue (Fig. 1a, see Table 1 for model selection results for Experiment I), with highest foraging rates in the high culling treatments. In contrast, crab removal and predator cue interactively affected the indirect effect of predators on oyster mortality (Fig. 1b, Table 1). Oyster mortality decreased with crab removal in the absence of fish cue, but it increased with crab removal when fish cue was present (Fig. 1b). Although overall oyster mortality was high in this experiment, crab foraging rates were constant over the course of the 4-day experiment (y = −8.72× +39.20; R^2^ = 0.99).

**Table 1:** Results of nested linear mixed-effect models for Experiment I.

Response variable	Model	df	dAIC	Weight
Per capita crab foraging rate	Foraging = Intercept + (Trial)	3	8.8	0.010
	**Foraging = Crab culling + (Trial)**	5	0.0	0.861
	Foraging = Crab culling + Predator cue + (Trial)	6	4.3	0.099
	Foraging = Crab culling * Predator cue + (Trial)	8	6.8	0.029
Oyster mortality	Mortality = Intercept + (Trial)	3	9.2	0.009
	Mortality = Crab culling + (Trial)	5	6.7	0.031
	Mortality = Predator cue + (Trial)	4	7.6	0.019
	Mortality = Crab culling + Predator cue + (Trial)	6	5.1	0.068
	Mortality = Crab culling * Predator cue + (Trial)	8	0.0	0.872
Non-consumptive effect size	**NCE = Intercept + (Trial)**	3	0.0	0.948
	NCE = Culling treatment + (Trial)	4	5.8	0.052
Consumptive effect size	**CE = Intercept + (Trial)**	3	0.0	0.945
	CE = Culling treatment + (Trial)	4	5.7	0.055
Density-mediated indirect interaction	**DMII = Intercept + (Trial)**	3	0.3	0.463
	DMII = Culling treatment + (Trial)	4	0.00	0.537

Bold indicates best model. Parentheses denote random effects. dAIC is the difference between the AICc of a particular model compared to the lowest AICc observed. The Akaike weight is calculated as the model likelihood normalized by the sum of all model likelihoods; values close to 1 indicate greater confidence in the selection of a model.

**Figure 2:**
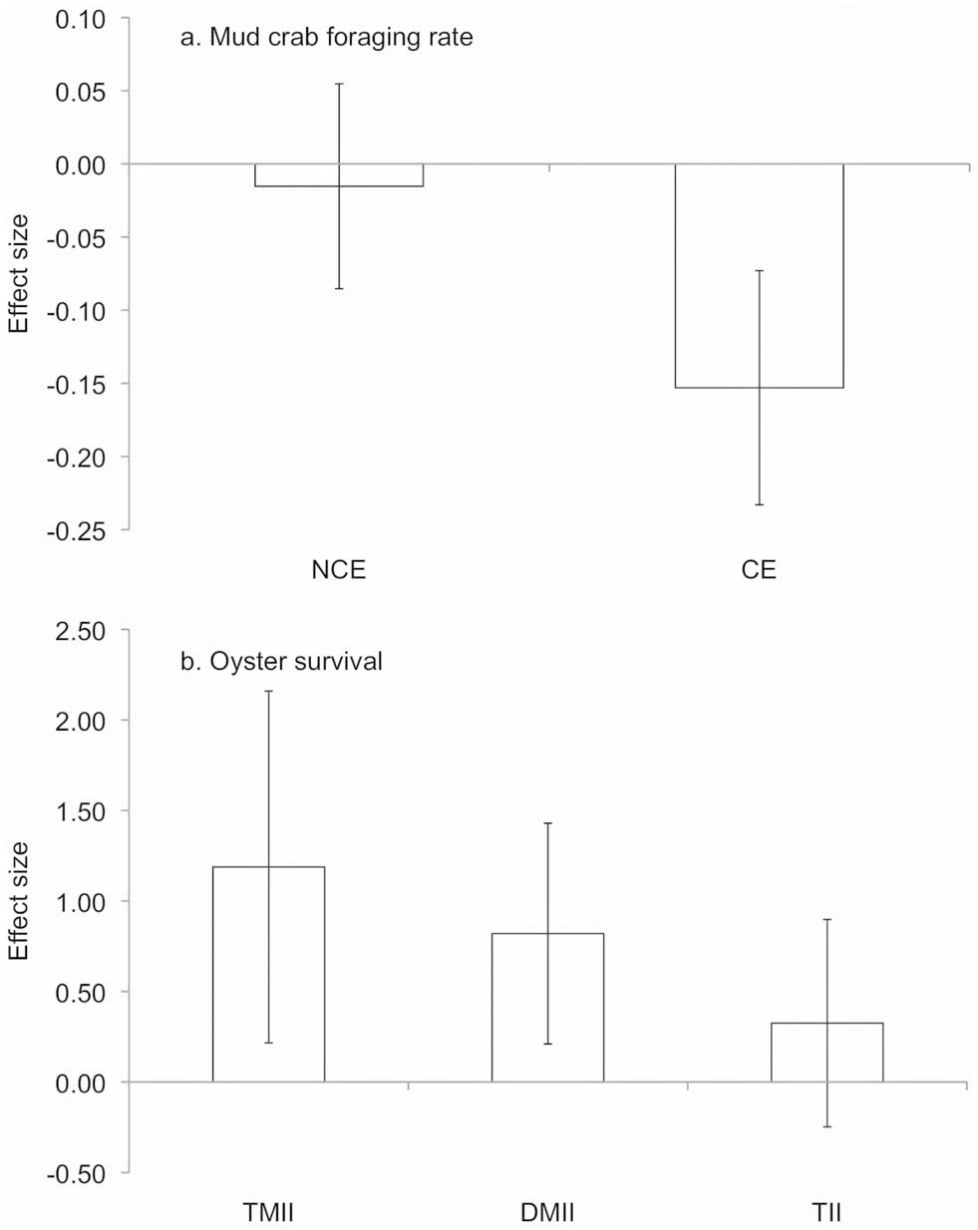
Direct and indirect predator effects in Experiment I. (a) The strength of non-consumptive (NCE) and consumptive (CE) effects on mud crab foraging rates in Experiment I. A negative effect size indicates that crab foraging rates were enhanced by predator cues (NCE) or crab removal (CE). Neither NCEs nor CEs varied by culling treatment (high cull or low cull). Bars represent means(±SE). (b) The strength of trait-mediated indirect interactions (TMII), density-mediated indirect interactions (DMII) and total indirect predator interactions (TII) on oyster abundance in Experiment I. A positive effect size indicates that oyster abundance was enhanced by predator cues (TMII), culling (DMII), or the combination of high culling and predator cues (TII). DMIIs did not vary by culling treatment (high cull or low cull). Bars represent means(±SE).

Calculating the effect size of consumptive and non-consumptive predator effects on crab foraging rates allowed us to compare the direction and strength of these direct interactions in a standardized manner. Consumptive effects (CEs) were negative, indicating that removal (culling) of crabs by simulated predation increased crab foraging rates (Fig. 2a). In contrast, non-consumptive predator effects (NCEs) were weak and not distinguishable from zero (Fig. 2a). Neither direct CEs nor NCEs differed significantly by culling treatment (Fig. 2a, Table 1). When we quantified the strength of each type of indirect predator effect on oysters, we found that both positive non-consumptive indirect effects (i.e., trait-mediated indirect interactions (TMIIs)) and positive consumptive indirect effects (i.e., density-mediated indirect interactions (DMIIs)) independently increased oyster abundances (Fig. 2b). DMIIs did not differ significantly between the high and low culling treatments (Table 1). In combination, TMIIs and DMIIs led to a negligible total indirect predator interaction (TII; Fig. 2b); oyster mortality was similar in the presence of predator cues and high culling as in the absence of predator cues and no culling (Fig. 1b).

**Figure 3:**
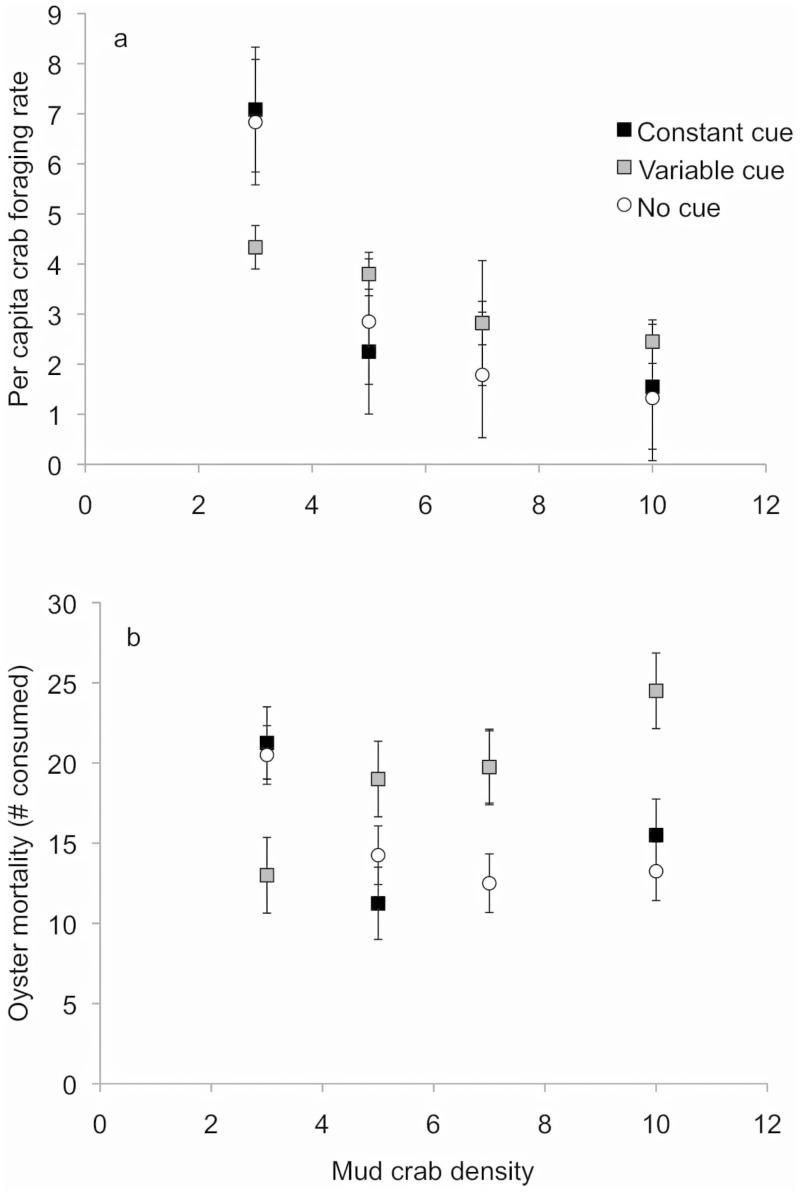
The effects of predator cue and mud crab density on (a) mud crab per capita foraging rate and (b) the number of juvenile oysters consumed during Experiment II. Closed squares indicate predatory fish cue is present at every high tide; gray squares indicate predator cue is present at every other night-time high tide; open circles indicate predator cue is absent at high tide. Symbols represent means(±SE). For reference, the average mud crab density in Experiment 1 was as follows: high culling = 7.3; low culling = 9.0; no culling = 10.0.

In Experiment II, crab density strongly influenced overall crab per capita foraging rates, with higher foraging rates at lower crab densities (Fig. 3a, see Table 2 for model selection results for Experiment II). Predator cue, whether constant or variable, did not impact per capita crab foraging rates. However, there was an interaction between predator cue treatment and crab density on oyster abundance at the end of the 4-day experiment (Fig. 3b, Table 2). Overall oyster mortality decreased linearly with crab density in the absence of predator cue (y = −0.95× +21.05; R^2^ = 0.60), increased linearly with crab density with variable predator cues (y = 1.52× +9.56; R^2^ = 0.93), and showed no relationship with crab density in the constant predator cue treatment.

**Table 2:** Results of nested linear mixed-effect models for Experiment II.

Response variable	Model	df	dAIC	Weight
Per capita crab foraging rate	**Foraging = Density**	3	0.0	0.900
	Foraging = Predator cue	4	17.7	<0.001
	Foraging = Density + Predator cue	5	4.8	0.081
	Foraging = Density * Predator cue	7	7.9	0.018
Oyster mortality	Mortality = 1+ (Day)	3	14.3	<0.001
	Mortality = Density + (Day)	4	5.0	0.048
	Mortality = Predator cue + (Day)	5	9.9	0.004
	Mortality = Predator cue + Density + (Day)	6	0.9	0.365
	Mortality = Predator cue * Density + (Day)	8	0.0	0.582

Bold indicates best model. Parentheses denote random effects. dAIC is the difference between the AICc of a particular model compared to the lowest AICc observed. The Akaike weight is calculated as the model likelihood normalized by the sum of all model likelihoods; values close to 1 indicate greater confidence in the selection of a model.

## Discussion

Consumptive predator effects (i.e., crab removal) resulted in increased per capita mud crab foraging rates in Experiment I (Fig. 2a): in the absence of predator cues, crab foraging rates were higher in the high culling treatment (i.e., lower density) than in the no culling treatment (i.e., higher density; Fig. 1a). These CEs were stronger than non-consumptive effects of predator cues on crab foraging rates, which did not differ significantly from zero (i.e., crab foraging rates in the absence of crab removal were similar with and without predator cues; Fig. 1a). We did not quantify the strength of the water-borne chemical cues in our predator cue treatments. However, we did detect effects of predator cues on oyster abundance in both experiments, suggesting that the slight NCEs in Experiment I are not an artifact of the use of water-borne cues.

In both of our experiments, per capita crab foraging rates were highly influenced by crab density: per capita foraging rates were higher at higher crab removal rates (and thus lower density) in Experiment I (Fig. 1a) and at lower crab densities in Experiment II (Fig. 3a). These results are consistent with previous research showing strong intra-specific interactions in this prey species [25]. However, our results contrast with the prediction that animals should eat faster in larger groups [26]. This pattern of lower overall foraging rates could result from several mechanisms that we were unable to address: (1) crabs uniformly reduce their foraging rates at high density due to antagonistic interactions with one another, or (2) an increase in feeding rates by a few crabs is outweighed by a reduction in feeding by competitively inferior crabs.

Prey density, which can vary in nature due to predation or other factors such as recruitment, interacted with predator cues to affect the basal resource (oysters) in this system. In Experiment I, this interaction resulted from contrasting effects of predator cue on oyster mortality in the no culling (high density) and high culling (low density) treatments (Fig. 1b). A similar pattern of oyster mortality was observed for the constant predator cue and no predator cue treatments in Experiment II over an equivalent range of crab density (7–10 crabs per mesocosm). However, the interaction between prey density and predator cues in Experiment II was caused by the variable cue treatment; crab foraging rates and oyster mortality in the constant and no predator cue treatments were similar over the larger range of crab density tested in this experiment (Fig. 3b). This similarity illustrates that changes in crab behavior due to conspecific interactions and predation threat are not additive, because reductions in foraging rate for a given crab density were equivalent in the presence and absence of predator cues. Instead, reductions in crab foraging in response to a high risk of predation may reduce interference interactions with conspecifics.

Higher per capita crab foraging (Fig. 3a) and higher oyster mortality (Fig. 3b) in the variable predator cue treatment than in the constant cue treatment for a given density conform with predictions from the risk allocation hypothesis [18], [21]. In accordance with this hypothesis, increases in foraging activity during risk-free periods of the variable predator cue treatment could have contributed to greater foraging overall. Alternatively, the level of risk exposure in the variable cue treatment may have sufficiently stressed the prey to increase metabolic demand and in turn elevate per capita foraging (compared to the same densities with no cue or constant cue; e.g., [27], [28], [29]). Regardless of the specific mechanisms underlying these patterns, our results emphasize that an understanding of variation in the natural predator cue environment is critical to testing and interpreting non-consumptive predator effects.

In contrast to the reduction in oyster mortality by mud crabs observed in previous studies of a similar tri-trophic oyster reef food web [12], [16], [17], oyster mortality in our experiment was higher in the presence of variable predator cues than in the absence of predator cues, except at very low crab densities (Fig. 3b). Several factors may have contributed to this divergence. First, the top fish predator tested in our experiment (hardhead catfish) differed from that tested in previous work (oyster toadfish). Although both fish species consume mud crabs, they differ in their utilization of reef habitats: toadfish are resident on many intertidal reefs, burrowing into the reef matrix at low tide, whereas catfish move on and off the reef with the tide (D. Kimbro, personal observation). Such differences in predator identity, and particularly predator foraging mode, can be important to the outcome of indirect predator effects [10], [11], [12].

Variation in predator identity between our experiments and previous studies is confounded with other differences that may influence the strength of predator effects. Although not quantified, variation in the abiotic environment (e.g., temperature) in the subtropical Gulf of Mexico vs. temperate North Carolina could have influenced organism metabolic rates and activity levels [30], [31]. Alternatively, mud crabs may exhibit regional variation in their predator response between the Atlantic reefs tested previously and the Gulf of Mexico reefs examined here, similar to geographic variation within species of consumers for particular prey (e.g., [32], [33]). Finally, variation in experimental methods likely created different predator cue environments across studies; as discussed below, slight changes in predator cues can have strong effects on consumer foraging. For instance, Grabowski and colleagues compared oyster reef mesocosms with toadfish individuals present to those with toadfish absent [12], [16], [17], whereas we tested the effects of water-borne predator cues only. The absence of visual predator cues in our study could have influenced crab responses [34]. In addition, we simulated a mixed tidal regime, with predator cues present only at high tides; reefs in past studies were submerged throughout the experiment. Finally, it is challenging to quantify the strength of the cues used in our study relative to previous studies, or to what crabs experience in the field. Teasing apart the effects of predator identity, abiotic conditions, consumer foraging modalities, predator cue environment, and experimental design on direct and indirect predator effects on oyster reef communities is an important next step.

Our experiments were relatively short (4 days) to prevent prey depletion of oysters. This brief duration may have led us to overestimate indirect predator effects, in that crabs may have not been sufficiently hungry over this relatively short duration to exhibit ‘risky’ behaviors when predator cues were present [19]. However, several lines of evidence suggest experimental duration was not important to our results. First, predator cues in the “constant” cue treatment were only present at high tide, providing daily predator-free periods in all mesocosms. Second, oyster consumption was higher in the presence of fish cues than the absence in the high culling treatment of Experiment I, and oyster consumption in the variable cue environment increased with density rather than decreased in Experiment II, suggesting that crabs were sufficiently hungry to forage even in the presence of predator cues. Third, mud crabs are notoriously ravenous and aggressive, often consuming one another within 24 hours when not fed sufficiently (R. Hughes, personal observation). Finally, the duration of our experiments were comparable to previous manipulations in a similar system (e.g., 6 days [17]), suggesting this time frame is reasonable for the species studied. Still, longer-term manipulations and surveys of field populations are needed to assess the generality of our findings.

We found that consumer density was the primary determinant of consumer per capita foraging rates, and it also had strong effects on basal resource abundance in concert with the predator cue environment. Thus, in systems in which consumer behavior is structured by strong intra-specific interactions such as the intertidal oyster reefs studied here [25], consumer density is likely to be important to the magnitude and direction of indirect predator effects (see also [24]). The low structural complexity of our experimental reefs may also have contributed to the importance of crab density and resulting intra-specific interactions: prior studies have shown that high structural complexity can alleviate interference interactions in oyster communities [12], [17], [25], [35].

The spatial and temporal characteristics of the predator environment can also have large impacts on prey effects [18], [20], [21], [22], [23]. Our study supports the assertion that relatively small increases in predator presence/consumer risk can have disproportionate effects on resource abundance (Fig. 3b; [20]). For instance, crabs in our variable predator cue treatment were exposed to predator cues for only 33% of the high tides in our experiment (in contrast to 0% in the no cue treatment, and 100% in the constant cue treatment), yet oyster abundance in the variable cue treatment consistently differed from the no cue treatment across the crab density gradient. Thus, previous manipulations that have used constant predator cues may have underestimated, rather than overestimated, the strength of trait-mediated indirect interactions on resource abundance. More importantly, we found a reversal in the sign of the relationship between prey density and resource availability in the variable cue treatment compared to either the constant predator cue or no cue treatments (Fig. 3b), highlighting the need to examine the direct effects of the predator risk environment not only on the prey, but also on their resources. Further, our results augment calls for explicit data regarding spatial and temporal variation in prey risk in natural populations in order to quantify accurately the non-consumptive effects of predators on prey behavior and resource abundance.

## Materials and Methods

### Study System

Intertidal oyster reefs along the northeastern Gulf of Mexico and southeast Atlantic coasts share a sub-web of residential species (i.e., a large portion of the food-web comprising highly interacting species that are found on reefs): dominant top predators include oyster toadfish (*Opsaunus* spp.), stone crabs (*Menippe mercenaria*), and blue crabs (*Callinectes sapidus*; [17], [36]); the most abundant intermediate consumers are mud crabs (Xanthidae; [37]); and 90% of the biomass on the basal trophic level is comprised of oysters (*Crassostrea virginica*; [17], [36], [38], [39]. In the northeastern Gulf of Mexico, the hardhead catfish (*Ariopsis felis*) is a common and abundant predator on oyster reefs that consumes mud crabs and other reef-associated invertebrates (D. Kimbro, unpublished data).

### Mesocosm Set-up

We conducted all experiments at the Florida State University Coastal and Marine Laboratory in St. Teresa, Florida, from June to September 2011 using an outdoor mesocosm array. Each experimental mesocosm consisted of a 78.5 L round plastic tub (diameter = 42 cm; area = 0.55 m^2^) with 2 drains: a 2.5 cm hole drilled 8.0 cm from the top of the tub prevented overflow of seawater at high tide, and a barbed reducer (1/4 in), 8.0 cm from the bottom allowed a gradual outflow of water. For all experiments, we simulated a semi-diurnal tidal schedule, with 2 high tides (11∶00 am–7∶00 pm; 11∶00 pm–8∶00 am) and 2 low tides (8∶00 am–11∶00 am; 7∶00 pm–11∶00 pm) daily.

We utilized a coupled, flow-through predator cue system similar to previous experimental manipulations testing non-consumptive predator effects (see, e.g., [4], [40], [41], [42]). All mesocosms received flow-through, sand-filtered seawater from the Gulf of Mexico via the seawater system at Florida State University Coastal and Marine Laboratory. Seawater for mesocosms receiving predator “cues” first circulated through a single 100 gallon flow-through tank (area = 1.04 m^2^) that housed two predators (hardhead catfish, *Ariopsis felis*). On a per unit area basis, this number of fish is slightly higher than the range found on local oyster reefs at high tide (0–10 fish per 3 m*3 m reef = 0–1.1/m^2^; D. Kimbro, unpublished data), but it was chosen to produce water-borne cues reflective of a localized area inhabited by catfish. The catfish were offered mud crabs as prey, and provided fresh shrimp *ad libidum* as an alternative food source during the experiment. The water was then pumped by a submersible utility pump affixed with a T-shaped PVC manifold with barbed reducers (3/8 in) through individual, clear vinyl tubes (1/2 in) to each predator cue mesocosm (mean (SE) flow rate = 2.57 (0.01) L/min). Mesocosms not assigned to a predator cue present treatment during high tide received seawater directly from the FSUCML seawater system at an equivalent flow rate (mean (SE) flow rate = 2.63 (0.01) L/min).

Prior to each experiment, we added 18.6 L (an approximate depth of 6 cm) of sieved sand to each mesocosm. Approximately 3 L of dead oyster shell was then placed on top of the sand in order to provide structure and habitat. Adult mud crabs (*Panopeus* spp.) greater than 20 mm carapace width were hand-collected from natural reefs in Apalachee Bay, FL, and added to the tanks. Juvenile oysters (*Crassostrea virginica*) less than 20 mm in shell diameter were also placed in each tank to serve as the basal resource for the crabs.

### Experiment I: Effects of Predator Cue and Crab Removal

In our first experiment, we tested the independent and interactive effects of fish predator cues (present or absent) and manual removal of crabs (i.e., “culling”; none, low or high; details provided below) on crab foraging rates and oyster mortality. Per capita crab foraging rates were calculated as the number of oysters consumed over the course of the experiment, divided by the number of days (4) and the average number of crabs present in each treatment. To increase our replication, we ran 2 separate trials of this experiment, with treatments randomly assigned to mesocosms for each trial. In trial 1, there were 3 replicate mesocosms of each predator treatment by crab removal combination. In trial 2, there were 5 replicate mesocosms of each predator treatment by crab removal combination. Mesocosms assigned to the predator cue treatment received seawater pumped from the catfish holding tank for 4 hours of the daily high tide (1∶00–5∶00 pm) and for the entire nighttime high tide (11∶00 pm–8∶00 am); mesocosms assigned to the no predator cue treatment received water from the primary water supply for the duration of each high tide.

Thirty juvenile hatchery-raised oysters [mean (SE) shell length = 15.36 (2.59) mm] were placed in each mesocosm prior to the addition of the crabs. Five oysters were affixed with marine adhesive (Z-spar) in a straight line to a thin strip of ceramic tile. Six tile strips were then placed into each mesocosm so that the oysters were vertically oriented.

Mesocosms each began with 10 crabs (mean (SE) carapace width in mm = 29.29(6.64)), which is within the range of natural densities on reefs in this area (D. Kimbro, unpublished data). In the no removal treatment, all crabs remained in the mesocosm for the 4-day duration of the experiment. In contrast, in the removal treatments crabs were haphazardly selected and removed manually following either a pre-set high (crab abundances days 1–4 = 10, 7, 5, 5; rate of decay = 0.27) or low (crab abundances days 1–4 = 10, 9, 8, 8; rate of decay = 0.08) daily removal schedule. These experimental removal rates were higher than natural rates of loss (R. Hughes, unpublished data), indicating that we likely over-estimate the relative importance of consumptive vs. non-consumptive effects. During each daily low tide, we quantified the number of live oysters on each tile and manually removed crabs according to each culling schedule.

### Experiment II: Effects of Predator Cue Variability and Crab Density

Because Experiment I and previous studies [25] indicated the importance of intra-specific interactions for crab foraging rates, our second experiment examined non-consumptive predator effects over a greater range of crab density (0, 3, 5, 7, or 10 crabs per mesocosm). In addition, we used our knowledge of predator behavior in this system to test multiple predator cue treatments: constant cues, variable cues, or no cues. Mesocosms assigned to the constant predator cue treatment received seawater pumped from the catfish holding tank for both the daily and nightly high tides. In contrast, mesocosms in the variable predator cue treatment received seawater from the primary system (with no predator cues) during each daily high tide and from the fish holding tank (with predator cues) on alternating nighttime high tides, beginning on the first night. This treatment was based on our surveys of predator fish assemblages on intertidal oyster reefs in our study region that show catfish are more often present at night (though they can be found on reefs during the day), and that they are not always found on the same reef over multiple sampled tides (R. Hughes, unpublished data). As in Experiment I, mesocosms assigned to the no predator cue treatment received water from the primary water supply for the duration of each high tide. There were 5 replicate mesocosms of each predator cue by density combination.

Fifty juvenile oysters (shell length = 5–15 mm) were added to each mesocosm in Experiment II. These oysters were collected from natural intertidal oyster reefs in Apalachicola Bay, FL, by selecting adult shells with 5–12 living juvenile *C.virginica* present. Before starting the experiment, 5–6 adult shells were added to each mesocosm, resulting in 50 juvenile oysters total. Each adult shell with juvenile oysters was marked with lacquer to be easily distinguished from the shell substrate in the mesocosm. We used a higher oyster density in this experiment, because overall levels of consumption were high in Experiment 1.

Crabs collected from local oyster reefs (mean(SE) carapace width in mm = 29.96(0.34)) were added to each mesoscosm according to the crab density treatments. During each daily low tide throughout the 4-day experiment, we quantified the number of live oysters on each adult shell.

### Statistical Analyses

For each of the mesocosm experiments, we used a model selection approach to assess a series of nested, mixed-effect models ranging from additive to interactive effects. We used the difference between the Akaike Information Criterion (AICc) of a particular model and the lowest AIC observed (the AIC difference, or dAIC) to determine which of the models best explained the observed data [43], [44]. We also calculated the Akaike weight as the model likelihood normalized by the sum of all model likelihoods, with values close to 1.0 indicating greater confidence in the model. If the dAIC score of a more complex model is greater than 2.0, then it is considered different from the next best model [45]. Candidate models and their dAIC scores and AIC weights are provided in tables. Analyses were conducted using the lmer function in the lme4 package and the AICctab function in the bblme package, R statistical software, version 2.11.1.

For Experiment I, we calculated effect sizes for the direct effects of predators on per capita prey foraging rates using a ratio-based approach as in [4]: To estimate the strength of consumptive effects (CEs), we calculated the ratio of per capita crab foraging rates in the crab removal treatments (high and low) to per capita crab foraging rates in the no crab removal treatment, all in the absence of predator cues. We examined the non-consumptive effect size (NCE) by calculating the ratio of the per capita crab foraging rates in the no crab removal treatment in the presence of predator cues to the per capita crab foraging rate in the no crab removal treatment in the absence of predator cues. A negative CE (see Results) indicates that crab removal increases crab foraging rates, whereas a negative NCE indicates that predator cues increase crab foraging rates.

We also calculated effect sizes for the indirect effects of predators on basal resource abundance following [4]. This approach has been shown to be the most consistent for determining these effects [46]. A density-mediated indirect interaction (DMII) effect size was calculated as the ratio of oyster abundance in the crab removal treatment in the absence of predator cues to oyster abundance in the no crab removal treatment in the absence of predator cues (with separate ratios for high removal and low removal). The trait-mediated indirect interaction (TMII) was calculated as the ratio of oyster abundance in the no removal treatment in the presence of predator cues to oyster abundance in the no removal treatment in the absence of predator cues. A positive DMII translates into a positive effect of crab removal on oyster abundance, and a positive TMII indicates a positive effect of predator cues on oyster abundance. Finally, we calculated the strength of the total indirect predator interaction (TII) by comparing the ratio of oyster abundance with high crab removal and predator cue to oyster abundance with no crab removal and no predator cue. We used model selection to determine whether culling treatment influenced the strength of either NCEs, CEs, or DMIIs on crab foraging rates.

### Ethics Statement

This study was carried out in strict accordance with the recommendations in the Guide for the Care and Use of Laboratory Animals of the National Institutes of Health. The protocol was approved by the Animal Care and Use Committee at the Florida State University (Permit Number: 1106).
